# A novel criterion of metabolically healthy obesity could effectively identify individuals with low cardiovascular risk among Chinese cohort

**DOI:** 10.3389/fendo.2023.1140472

**Published:** 2023-05-26

**Authors:** Qiyu Li, Pengbo Wang, Rui Ma, Xiaofan Guo, Yingxian Sun, Xingang Zhang

**Affiliations:** Department of Cardiology, The First Hospital of China Medical University, Shenyang, Liaoning, China

**Keywords:** metabolically healthy obesity, cardiovascular disease, risk factor, blood pressure, epidemiology

## Abstract

**Background and objective:**

Obesity has become a serious public health problem and brings a heavy burden of cardiovascular disease. Metabolically healthy obesity (MHO) is defined as individuals with obesity with no or only minor metabolic complications. Whether individuals with MHO have a lower cardiovascular risk remains controversial. In this study, a new criterion was used to define MHO and assess its predictive value for cardiovascular events and death. At the same time, the new criterion and the traditional criterion are compared to analyze the differences between different diagnostic criteria.

**Methods:**

A prospective cohort was established in northeast rural China from 2012 to 2013. Follow-up was conducted in 2015 and 2018 to investigate the incidence of cardiovascular events and survival. Subjects were grouped according to the metabolic health and obesity status. Kaplan-Meier curves were drawn to describe the cumulative risk of endpoint events in the four groups. Cox regression analysis model was constructed to evaluate the risk of endpoint events. Analysis of variance and *post hoc* analyses were used to calculate and compare differences in metabolic markers between MHO subjects diagnosed by novel and traditional criteria.

**Results:**

A total of 9345 participants 35 years of age or older without a history of cardiovascular disease were included in this study. After a median follow-up of 4.66 years, the data showed that participants in the MHO group had no significant increase in the risk of composite cardiovascular events and stroke, but had a 162% increase in the risk of coronary heart disease (HR: 2.62; 95%CI: 1.21-5.67). However, when using conventional criteria for metabolic health, mMHO group had a 52% increase in combined CVD risk (HR: 1.52; 95%CI: 1.14-2.03). By comparing the differences of metabolic indicators between MHO subjects diagnosed by the two criteria, MHO subjects diagnosed by the new criterion had higher WC, WHR, TG, FPG, and lower HDL-C levels except for lower blood pressure, showing more exposure to cardiovascular risk factors.

**Conclusions:**

The risk of combined CVD and stroke was not increased in MHO subjects. The new metabolic health criterion is superior to the traditional criterion and can effectively identify individuals with obesity with a lower risk of combined CVD. Blood pressure levels may be responsible for the inconsistent risk of combined CVD in MHO subjects diagnosed with both criteria.

## Introduction

The prevalence of obesity has nearly tripled in the past 40 years, presenting a global epidemic trend. By 2016, more than 1.9 billion adults were overweight and more than 650 million of them were with obesity, generating a huge health burden (1).The prevalence of cardiovascular disease (CVD) has also grown from 271 million in 1990 to 523 million in 2019 (2), becoming one of the leading causes of disability and mortality. Obesity is considered as an independent risk factor for atherosclerosis, ischemic heart disease and strokes (3). Previous studies have shown the strong correlation between obesity and heart metabolic risk (4). Moreover, complications of obesity, such as type 2 diabetes, non-alcholic fatty liver disease and sleep apnea are also risk factors for CVD (5).

Individuals with obesity are more likely to have metabolic disorders, which further increase their risk of cardiovascular disease (6). However, not all patients with obesity suffer from metabolic disorders that manifest as metabolic health, known as metabolically healthy obesity (MHO) (7) (8). Some scholars believe that individuals with MHO do not have an increased risk of cardiovascular events (9, 10). However, some studies have shown that MHO status is associated with adverse cardiovascular outcomes (5, 11–15). Whether individuals with MHO have a lower cardiovascular risk is currently a matter of debate, largely due to the lack of a clear and unified definition of MHO. At least 30 different criteria have been applied in previous studies (16), which makes the prevalence of MHO vary widely, ranging from 4.2% to 51% (17–19). Some researchers believe that assessing the risk of MHO individuals largely depends on how to define MHO (18), so finding an effective method to identify people with obesity with real low cardiovascular risk is very important for cardiovascular prevention.

Recently, Zembic et al. proposed a more reliable new definition of MHO through scientific calculation based on the data of a large population cohort, and verified its effective value in predicting the risk of cardiovascular death and all-cause death in patients with obesity in Western populations (20). This study used this new MHO criterion to define metabolic health, validated it in the Asian population, and further explored its predictive value for cardiovascular disease. At the same time, the MHO population defined by the new criteria and the MHO population defined by the traditional criteria were compared at the metabolic level to find differences between MHO subjects under the different definitions.

## Materials and methods

### Study population

Subjects in this study were 11,956 residents aged ≥35 in Liaoning province of China who participated in the Northeast Rural Cardiovascular Health Study (NCRCHS) from January 2012 to August 2013, details had been described elsewhere (21, 22). In brief, by the method of multi-stage random cluster sampling, 26 villages were randomly selected from 3 counties of Liaoning Province, all residents aged ≥35 in the villages were invited to participate and the ones who completed baseline investigation were enrolled. At baseline, information about the subjects was collected through questionnaires, blood biochemical tests, and physical measurements. Two follow-up visits were conducted in 2015 and 2018 to collect information on cardiovascular events and deaths during this period. Of those enrolled, 10,349 completed at least one follow-up visit. This study was approved by the Ethics Committee of China Medical University (Shenyang, China) and all participants gave written informed consent. All methods were performed in accordance with the relevant guidelines and regulations.

**Figure 1:**
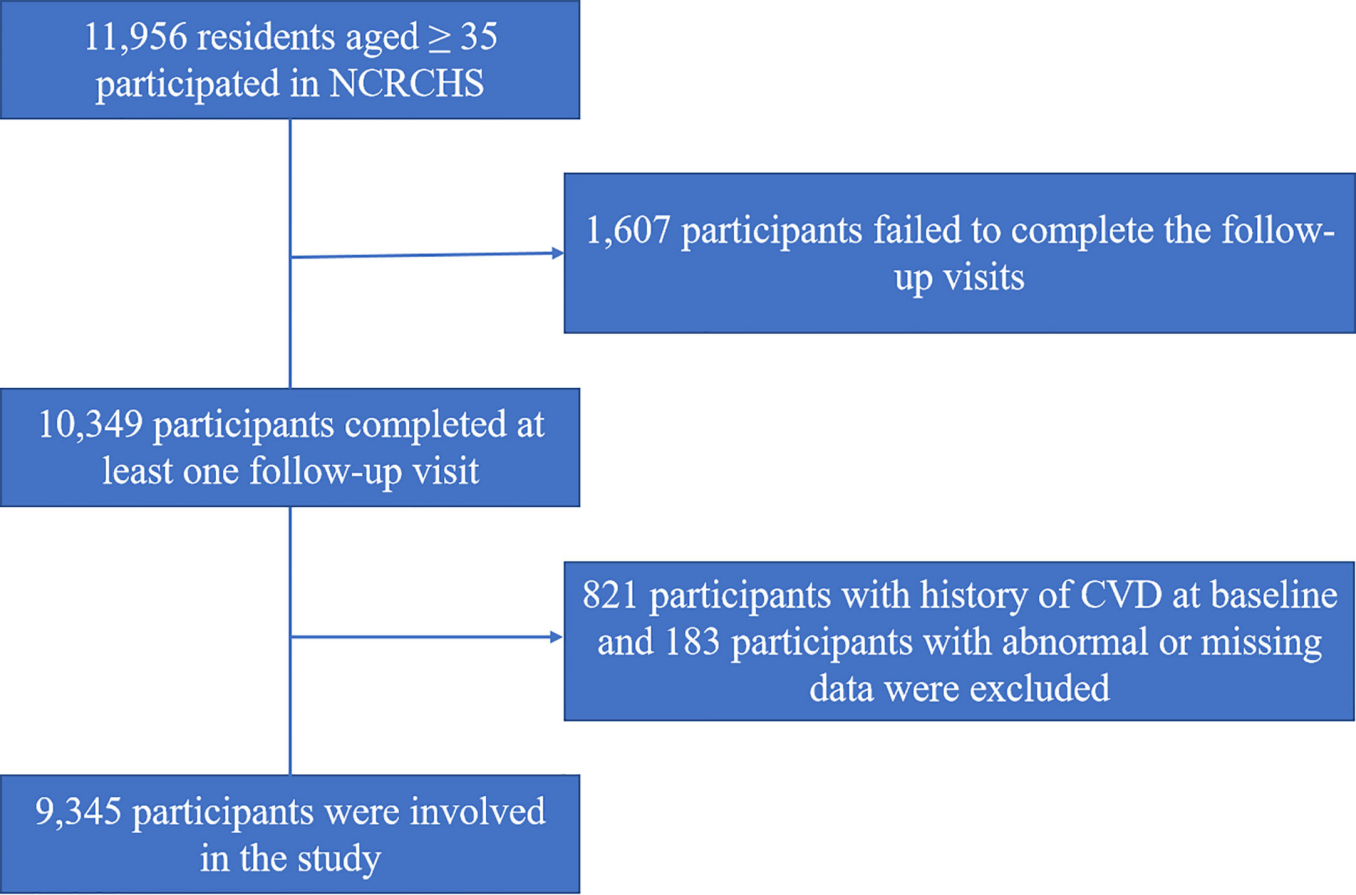
Flow chart of the subject screening process.

Participants with history of CVD at baseline (n=821) and those with abnormal or missing data (n=183) were excluded. Eventually, a total of 9345 participant data were available. The screening procedure is illustrated in **Figure 1**.

### Study variables

Information on age, sex, smoking and drinking status, hypertension, diabetes, and current medication use were recorded through face-to-face questionnaires. Stroke and CHD history at baseline were self-reported and confirmed by medical records. Blood pressure was measured by trained personnel using standard electronic sphygmomanometers (HEM-741C; Omron, Tokyo, Japan) in a quiet room with appropriate temperature. Participants were asked to rest adequately for at least five minutes prior to the measurement, then place their bare upper arms at the level of the heart. Blood pressure was measured 3 times, 2 minutes apart, and the average of the recordings was used for the final analysis. Weight and height were measured with participants wearing light clothing and no shoes. The measurements were accurate to 0.1 kg and 0.1 cm. Then measure waist circumference (WC) at the umbilicus and horizontal hip circumference (HC) at the most prominent part of the buttocks using a tape measure. All participants were required to fast for at least 12 hours beforehand, and their intravenous blood was collected at the elbow the following morning. The blood sample was added to a vacuum tube containing anticoagulant and the plasma was obtained by centrifugation. Fasting blood glucose (FPG), serum creatinine (Scr), uric acid (UA), triglyceride (TG), total cholesterol (TC), low density lipoprotein cholesterol (LDL-C), high density lipoprotein cholesterol (HDL-C) and some other biochemical indicators were automatically analyzed by the machines (Olympus AU 640, Tokyo, Japan). Body Mass Index (BMI) was calculated as weight (kg) divided by height (meters) squared. Waist-to-hip ratio (WHR) was calculated as WC divided by HC. Estimated glomerular filtration rate (eGFR) was calculated using the Chronic Kidney Disease Epidemiology Collaborative Equation (23).

### Definitions of metabolic health and obesity status

Obesity was defined as BMI ≥25kg/m_2_ according to the Asia-Pacific criteria (24). For the definition of metabolic health, the novel criterion proposed by Zembic A et al (20) was used in this study: subjects who met all 3 of the following criteria were considered as metabolically healthy: (a) systolic blood pressure <130mmHg and no current antihypertensive medication use; (b) WHR <1.03 for male or <0.95 for female respectively; (c) no diabetes. In this study, diabetes was defined as having a self-reported history of diabetes or taking antidiabetic drugs at present or FPG ≥7.0mmol/L (25). Another traditional criterion to define metabolic health was based on the absence of metabolic syndrome proposed by the harmonized International Diabetes Federation criteria (26). Metabolic syndrome (Mets) was defined as a combination of 3 or more of the following 5 criteria: (a) WC ≥85 cm for males and ≥80 cm for females; (b) BP ≥130/85 mmHg or current use of antihypertensive drugs; (c) FPG ≥5.6 mmol/L or current use of antihyperglycemic agents; (d) TG ≥1.7 mmol/L; (e) HDL-C <1.0 mmol/L for males and <1.3 mmol/L for females.

Metabolic health and obese status were combined to divide all participants into four groups: (a) metabolically healthy non-obesity (MHN), defined as BMI <25 kg/m_2_ and metabolically healthy; (b) metabolically unhealthy non-obesity (MUN), defined as BMI <25 kg/m_2_ and metabolically unhealthy; (c) Metabolically healthy obesity (MHO), defined as BMI ≥25 kg/m_2_ and metabolically healthy; (d) metabolically unhealthy obesity (MUO), defined as BMI ≥25 kg/m_2_ and metabolically unhealthy.

Subsequently, in a similar manner, the population was re-divided into four different groups: mMHN, mMUN, mMHO, and mMUO based on the presence or absence of Mets and obese status. mMHN stands for metabolically healthy (without MetS) individuals without obesity; mMUN stands for metabolically unhealthy (with MetS) individuals without obesity; mMHO stands for metabolically healthy (without MetS) individuals with obesity; mMUO stands for metabolically unhealthy (with MetS) individuals with obesity.

### Endpoints

The primary outcome was incident CVD which included fatal and non-fatal stroke and CHD (27).The secondary outcomes of interest were separate stroke and CHD incidence, cardiovascular death and all-cause mortality. CHD was defined as a diagnosis of hospitalized angina, myocardial infarction, undergoing revascularization, or death from coronary artery related disease (28). Stroke was defined as rapidly developing signs of focal or global disturbance of cerebral function, lasting more than 24 h (unless interrupted by surgery or death) with no apparent non-vascular cause according to the World Health Organization Multinational Monitoring of Trends and Determinants in Cardiovascular Disease criteria (29, 30). All available clinical information, including medical records and death certificates, was collected and independently reviewed and adjudicated by an endpoint evaluation committee.

### Statistical analysis

Descriptive statistics were first performed for variables, with continuous variables presented as means and standard deviations and categorical variables as numbers and percentages. Non-parametric tests and chi-squared tests were used to assess differences between groups. The Kaplan-Meier method was used to estimate the cumulative risk of endpoint events among participants in different groups, and the log-rank test was used to compare the differences between groups. Hazard ratios (HRs) and 95% confidence intervals (CI) were then calculated using the Cox proportional hazards model with the MHN group as the reference. The adjusted variables were diagnosed using collinearity, and variance inflation factor (VIF) <10 was considered acceptable. Variance analysis and *post hoc* analysis were used to compare whether there were differences in metabolic parameters between the MHO groups defined by the two criteria. All data analyses above were performed using IBM SPSS statistical software, version 26 (IBM Corporation, Armonk, New York, USA), and a two-tailed P value of less than 0.05 was considered statistically significant.

## Results

### Baseline characteristics of the study population

The 9345 participants were divided into MHN, MUN, MHO and MUO groups based on baseline metabolic health and obesity status. Of all participants, 31.6% were metabolically healthy and 44.3% were with obesity. The baseline characteristics of the participants are listed in **Table 1**. At baseline, 2069 participants (22.1%) were MHN, 3132 (33.5%) MUN,886 (9.5%) MHO, and 3258 (34.9%) MUO. Among 4144 subjects with obesity, the proportion of metabolically healthy subjects was 21.4%. Participants who were metabolically unhealthy, whether with obesity or not, had a higher proportion of male and older adults, lower household incomes, a higher proportion of people with a family history of stroke, and higher rates of smoking, alcohol consumption, and regular exercise than participants who were metabolically healthy. Compared to the MHN group, subjects in the MHO group had higher SBP, DBP, TG, TC, LDL-C, UA and FPG but significantly lower than those in the MUO group, while HDL-C and eGFR were lower but also higher than those in the MUO and MUN groups. In addition, BMI was also higher in the MUO group than in the MHO group.

**Table 1:** Baseline characteristics of subjects grouped according to metabolic health and obesity status.

	MHN (n=2069)	MUN (n=3132)	MHO (n=886)	MUO (n=3258)	p-Value
age (year)	49.1 ± 9.2	56.5 ± 10.5	48.0 ± 8.4	53.9 ± 10.0	<0.001
male (%)	865 (41.8)	1607 (51.2)	355 (40.0)	1561 (47.9)	<0.001
ethnicity of Han (%)	1953 (94.3)	2981 (95.1)	830 (93.6)	3050 (93.5)	0.089
family income>5000CNY (%)	1895 (91.5)	2680 (85.5)	825 (93.0)	2923 (89.6)	<0.001
family history of CHD (%)	276 (13.3)	401 (12.7)	125 (14.1)	479 (14.7)	0.15
family history of Stroke (%)	275 (13.3)	519 (16.5)	128 (14.4)	596 (18.3)	<0.001
current smoking (%)	778 (37.6)	1300 (41.5)	266 (30.0)	1016 (31.2)	<0.001
current drinking (%)	393 (19.0)	876 (27.9)	166 (18.7)	779 (23.9)	<0.001
regular exercise (%)	346 (16.7)	649 (20.7)	158 (17.8)	741 (22.7)	<0.001
BMI (kg/m^2^)	21.9 ± 1.9	22.4 ± 1.8	27 ± 2.8	28.1 ± 2.7	<0.001
WC (cm)	74.6 ± 6.5	77.7 ± 7.1	86.5 ± 7.2	89.7 ± 8.0	<0.001
WHR	0.81 ± 0.06	0.84 ± 0.08	0.87 ± 0.06	0.89 ± 0.07	<0.001
SBP (mm Hg)	118 ± 8	150 ± 19	120 ± 7	153 ± 20	<0.001
DBP (mm Hg)	72 ± 7	85 ± 11	74 ± 7	87 ± 11	<0.001
TC (mmol/L)	4.9 ± 1.0	5.3 ± 1.1	5.1 ± 1.0	5.4 ± 1.1	<0.001
TG (mmol/L)	1.2 ± 1.0	1.4 ± 1.3	1.6 ± 1.4	2.0 ± 1.7	<0.001
LDL-C (mmol/L)	2.6 ± 0.7	2.9 ± 0.8	2.9 ± 0.8	3.2 ± 0.9	<0.001
HDL-C (mmol/L)	1.4 ± 0.4	1.5 ± 0.4	1.3 ± 0.3	1.3 ± 0.4	<0.001
UA (μmmol/L)	269 ± 76	280 ± 79	289 ± 80	305 ± 88	<0.001
eGFR (ml/min/1.73m^2^)	96.7 ± 12.7	92.9 ± 16.4	96.5 ± 13.7	93.2 ± 15.3	<0.001
FPG (mmol/L)	5.3 ± 0.5	5.9 ± 1.7	5.4 ± 0.5	6.2 ± 1.9	<0.001

CNY, China Yuan; CHD, coronary heart disease; BMI, body mass index; WC, waist circumference; WHR, waist hip rate; SBP, systolic blood pressure; DBP, diastolic blood pressure; TC, total cholesterol; TG, triglyceride; LDL-C, low-density lipoprotein cholesterol; HDL-C, high-density lipoprotein cholesterol; eGFR, estimated glomerular filtration rate; FPG, fasting plasma glucose;

MHN, metabolically healthy non-obesity; MUN, metabolically unhealthy non-obesity; MHO, metabolically healthy obesity; MUO, metabolically unhealthy obesity.

Data are expressed as M ± SD or as n (%). Nonparametric tests and chi-square tests were used to assess the differences.

### Comparison of cumulative risk of endpoint events in the four groups

During a median follow-up of 4.66 years, 464 composite cardiovascular events and 342 all-cause deaths occurred, including 316 strokes, 160 coronary heart disease events, and 160 fatal cardiovascular events. Among the participants, 5.0% had composite cardiovascular disease (1.7% had fatal cardiovascular events), 3.4% had stroke, 1.7% had coronary heart disease, and 3.7% had all-cause mortality.

**Figure 2:**
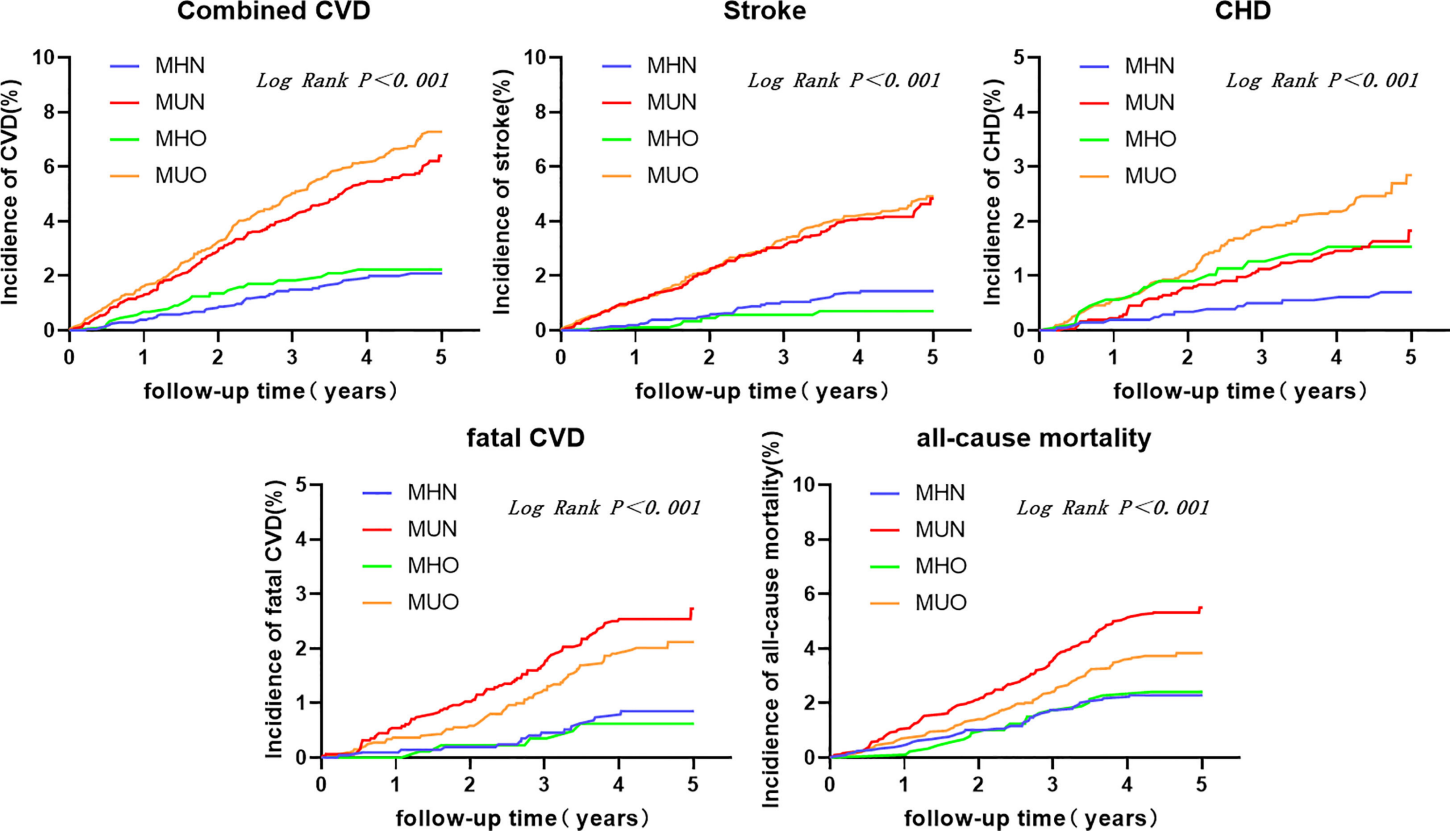
Kaplan-Meier curves of metabolic obesity status and cumulative risk of end-point events.

The study used composite cardiovascular events, stroke, CHD, fatal CVD, and all-cause mortality as key events, respectively. The Kaplan-Meier curves are plotted to describe the cumulative risk of endpoint events for the four groups of subjects are shown in **Figure 2**. The results of the study with composite cardiovascular events as end-point events are shown in **Figure 2A**. Figureshows that the MHN group has the lowest cumulative risk of occurrence, with a 5-year cumulative risk of less than 2%. Compared with the MHN group, the cumulative risk of MUN and MUO groups was significantly increased, and the 5-year cumulative risk was about 6%-8%, while the cumulative risk of the MHO group was not significantly different from the MHN group, the two curves were basically equal, and the difference between the groups was significant, Log-Rank < 0.001. For stroke, fatal CVD, and all-cause mortality, the results were similar, with little difference in cumulative risk between the MHN and MHO groups and varying degrees of increased risk in the MUN and MUO groups (**Figures 2B, D, E**). The results were slightly different for CHD, where the MHN group still had the lowest cumulative risk; in contrast, the MHO and MUN groups had a similar increase in the cumulative risk, and the MUO group had the highest increase in the cumulative risk (**Figure 2C**).

### Constructing Cox proportional hazard models to compare the risk of endpoint events

Incidence rates and Cox regressions were calculated separately for combined CVD, stroke, CHD, fatal CVD, and all-cause mortality from any cause. The hazard ratio and 95% confidence interval after the stepwise regression are shown in **Table 2**. Three regression models were constructed, with adjustment for age, sex, and ethnic Han status (yes or no, binary variable) in model 1. Model 2 was adjusted for current smoking (yes or no, binary variable), current alcohol consumption (yes or no, binary variable), annual family income (continuous variable), regular exercise or physical labor (yes or no, binary variable), family history of stroke (yes or no, binary variable), and family history of CHD (yes or no, binary variable) based on Model 1. Model 3 was further adjusted for eGFR, UA, and LDL-C based on Model 2.

**Table 2:** Incidence of the four groups of subjects and results of Cox regression analysis.

	MHN (n=2069)	MUN (n=3132)	MHO (n=886)	MUO (n=3258)
combined CVD	42 (2.03)	181 (5.78)	19 (2.14)	222 (6.81)
model 1	1.00 (ref)	1.64 (1.17-2.32)	1.19 (0.69-2.05)	2.40 (1.72-3.35)
model 2	1.00 (ref)	1.64 (1.17-2.32)	1.23 (0.72-2.12)	2.48 (1.77-3.46)
model 3	1.00 (ref)	1.63 (1.16-2.31)	1.15 (0.67-1.99)	2.28 (1.62-3.21)
stroke	28 (1.35)	134 (4.28)	6 (0.68)	148 (4.54)
model 1	1.00 (ref)	1.75 (1.16-2.66)	0.57 (0.24-1.38)	2.31 (1.54-3.48)
model 2	1.00 (ref)	1.70 (1.12-2.58)	0.60 (0.25-1.45)	2.36 (1.57-3.56)
model 3	1.00 (ref)	1.71 (1.12-2.59)	0.56 (0.23-1.35)	2.17 (1.43-3.31)
CHD	15 (0.72)	51 (1.63)	13 (1.47)	81 (2.49)
model 1	1.00 (ref)	1.56 (0.85-2.87)	2.73 (1.27-5.86)	2.97 (1.66-5.32)
model 2	1.00 (ref)	1.60 (0.88-2.93)	2.78 (1.29-5.97)	3.15 (1.76-5.63)
model 3	1.00 (ref)	1.55 (0.85-2.85)	2.62 (1.21-5.67)	2.90 (1.60-5.26)
fatal CVD	16 (0.77)	76 (2.43)	5 (0.68)	63 (1.93)
model 1	1.00 (ref)	1.49 (0.86-2.57)	0.89 (0.32-2.42)	1.56 (0.90-2.71)
model 2	1.00 (ref)	1.54 (0.89-2.67)	0.93 (0.34-2.54)	1.66 (0.95-2.89)
model 3	1.00 (ref)	1.51 (0.87-2.62)	0.90 (0.33-2.47)	1.55 (0.88-2.75)
all-cause mortality	45 (2.17)	160 (5.11)	20 (2.26)	117 (3.59)
model 1	1.00 (ref)	1.24 (0.89-1.75)	1.21 (0.71-2.05)	1.10 (0.78-1.56)
model 2	1.00 (ref)	1.28 (0.91-1.80)	1.24 (0.73-2.11)	1.15 (0.81-1.63)
model 3	1.00 (ref)	1.25 (0.88-1.76)	1.16 (0.68-1.97)	1.04 (0.72-1.49)

By comparing the incidence of endpoint events among the four groups, it was found that the MHN and MHO groups had lower incidence, while the MUN and MUO groups had significantly higher incidence than the former two groups. As an example of combined CVD, the MHN and MHO groups had an incidence of 2.03% and 2.14%, respectively, while the MUN and MUO groups had an incidence of 5.78% and 6.81%, respectively. For stroke, fatal CVD, and all-cause mortality from any cause, the outcome differences in incidence were similar. In CHD, however, the results were different, with an incidence of 0.72% in the MHN group, 1.63% and 1.47% in the MUN and MHO groups, respectively, and 2.49% in the MUO group.

For the results of Cox regression analysis, the results showed that in combined CVD, stroke, and CHD, the trend of results from the three models was stable, and the results showed that MUN and MUO were independent risk factors for combined CVD and stroke, while MHO and MUO were independent risk factors for CHD. After model 3 adjustment for all confounding factors, the risk of a combined CVD was increased by 63% in the MUN group compared with the MHN group (HR:1.63; 95%CI: 1.16-2.31), and the risk increased by 128% in the MUO group (HR:2.28; 95%CI: 1.62-3.21). However, the MHO group did not show a higher risk than the MHN group, the difference was not statistically significant (HR:1.15; 95%CI: 0.67-1.99). Similar results were found when stroke was used as the endpoint event. In model 3, the HR and 95%CI of the MUN and MUO groups were 1.71 (95%CI: 1.12-2.59) and 2.17 (95%CI:1.43-3.31), respectively. And 0.56 (95%CI:0.23-1.35) in MHO group. For CHD, the MHO group had a 162% increased risk (HR:2.62; 95%CI:1.21-5.67), and the risk increased by 190% in the MUO group (HR:2.90; 95%CI:1.60-5.26). However, there was no significant risk increasing in the MUN group (HR: 1.55; 95%CI: 0.85-2.85). No significant differences in the risk of fatal CVD and all-cause mortality were observed between the four groups in the three models.

**Figure 3:**
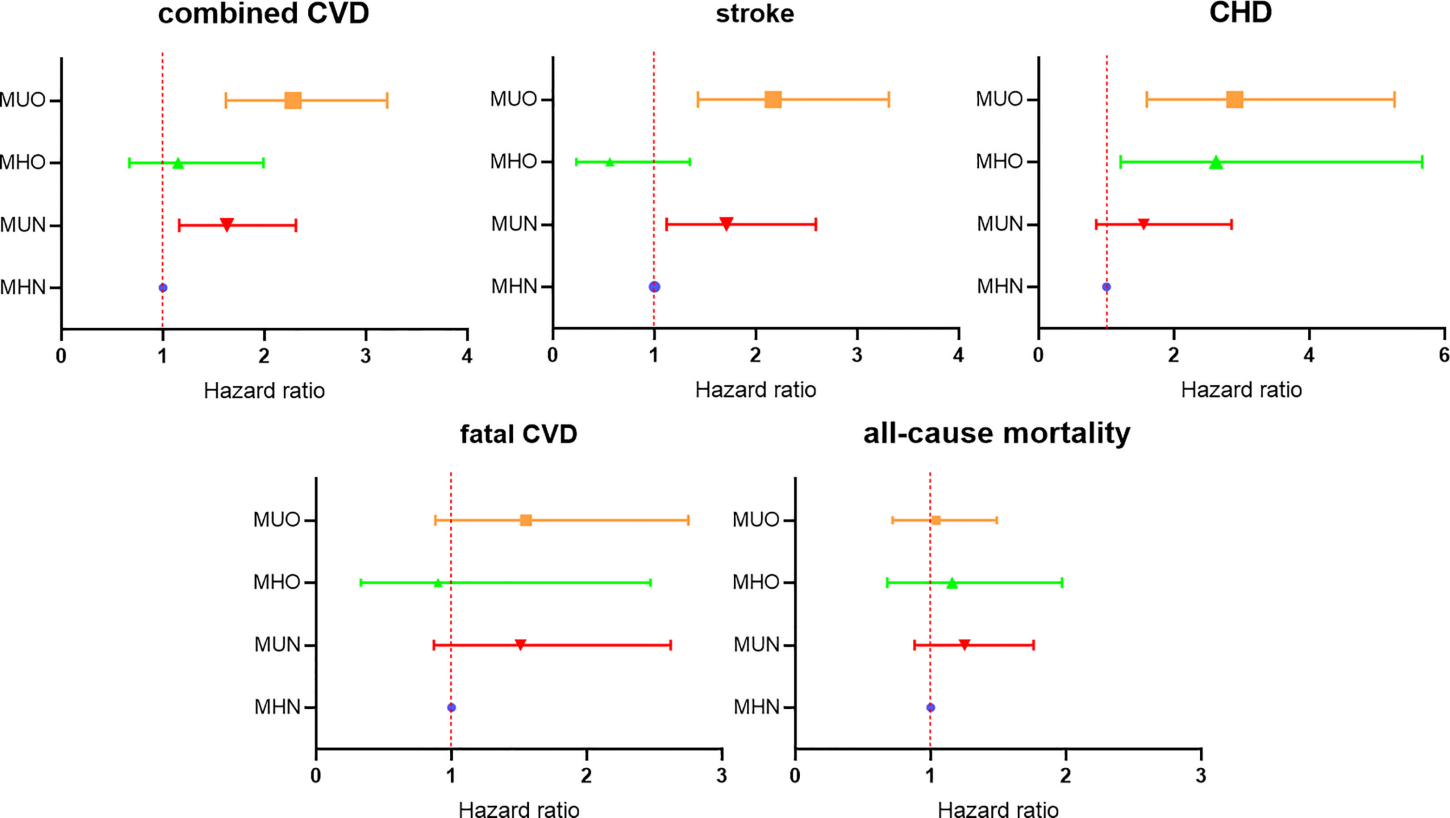
Comparison of the risk among the four groups based on Cox regression model 3.

Based on the results of Model 3, a forest plot was drawn to visually show the risk comparison among the four groups of subjects, as shown in **Figure 3**.

### Comparing the risk of combined CVD among the four groups defined by traditional criterion

Metabolic health was diagnosed according to traditional criteria (i.e., the presence or absence of metabolic syndrome) and was categorized into mMHN, mMUN, mMHO, and mMUO groups in combination with obesity status. The baseline characteristics are given in **Supplementary Table 1**. Compared with the new criteria, the proportion of metabolically healthy people defined by the traditional criteria was higher (60.4% vs. 31.6%), and the proportion of mMHO was also higher than that of MHO (16.4% vs. 9.5%).

**Figure 4:**
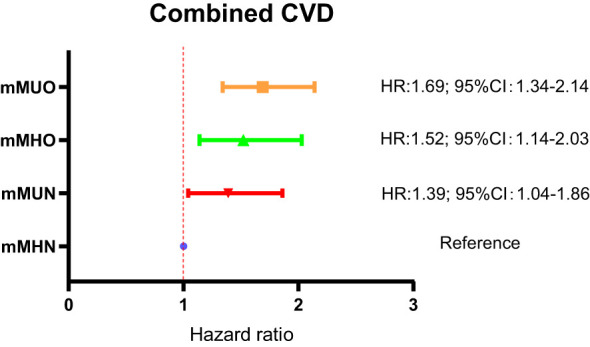
Comparison of combined CVD risk among the four groups of subjects defined by traditional criterion for metabolic health.

Subsequently, the Cox regression analysis was repeated for the four groups of subjects with composite cardiovascular events as end-point events, corrected for the confounding included in model 3, and a forest plot was plotted to show the risk comparison between the groups, as shown in **Figure 4**. The results showed a significant increase in combined CVD risk in the mMHO group, with a 52% increase in risk compared with the mMHN group (HR: 1.52; 95%CI: 1.14-2.03). Combined CVD risk was also increased in both mMUN and mMUO groups, with HR and 95%CI of 1.39 (1.04-1.86) and 1.69 (1.34-2.14), respectively.

### Comparison of combined CVD risk between MHO groups defined by the two criteria​

**Figure 5:**
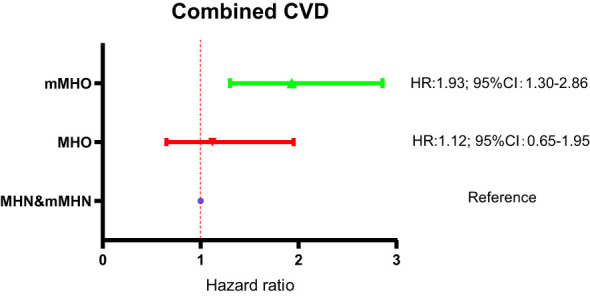
Comparison of combined CVD risk between MHO groups defined by the two criteria.

The risks of combined CVD in the MHO and mMHO groups had been described in **Figure 3A** and **Figure 4**, respectively, and reached inconsistent conclusions that the risk of the mMHO group was higher than that of the reference mMHN group, while the risk of the MHO group was not significantly different from that of the MHN group. To harmonize the reference standards and make further comparisons, the following analysis selected the MHN&mMHN group (patients who met both the new and traditional criteria for MHN) as the reference and performed separate Cox regression analyses (adjusted for the confounding factors included in model 3). The results showed that compared with the MHN&mMHN group, the mMHO group had a 93% increased risk (HR: 1.93; 95%CI: 1.30-2.86), while there was no increased risk in MHO group (95%CI: 0.65-1.95). The forest plot and the associated data are shown in **Figure 5**.

### Comparison of differences in metabolic indices between MHO groups defined by the two criteria

The above data shows that the CVD risk is different between the MHO and mMHO groups defined by the two different criteria. In order to further clarify the reasons, the following analysis compared the metabolic indicators (WC, WHR, SBP, DBP, TG, HDL-C, FPG) involved in the two criteria. Subjects were divided into MHO′, mMHO′and MHO&mMHO groups based on single or both diagnostic criteria. Variance and *post hoc* analyses were performed to compare the differences between the three groups and between the MHO′ and mMHO′ groups. The calculated results showed that P value < 0.001, indicating that the differences between the groups were significant.

**Figure 6:**
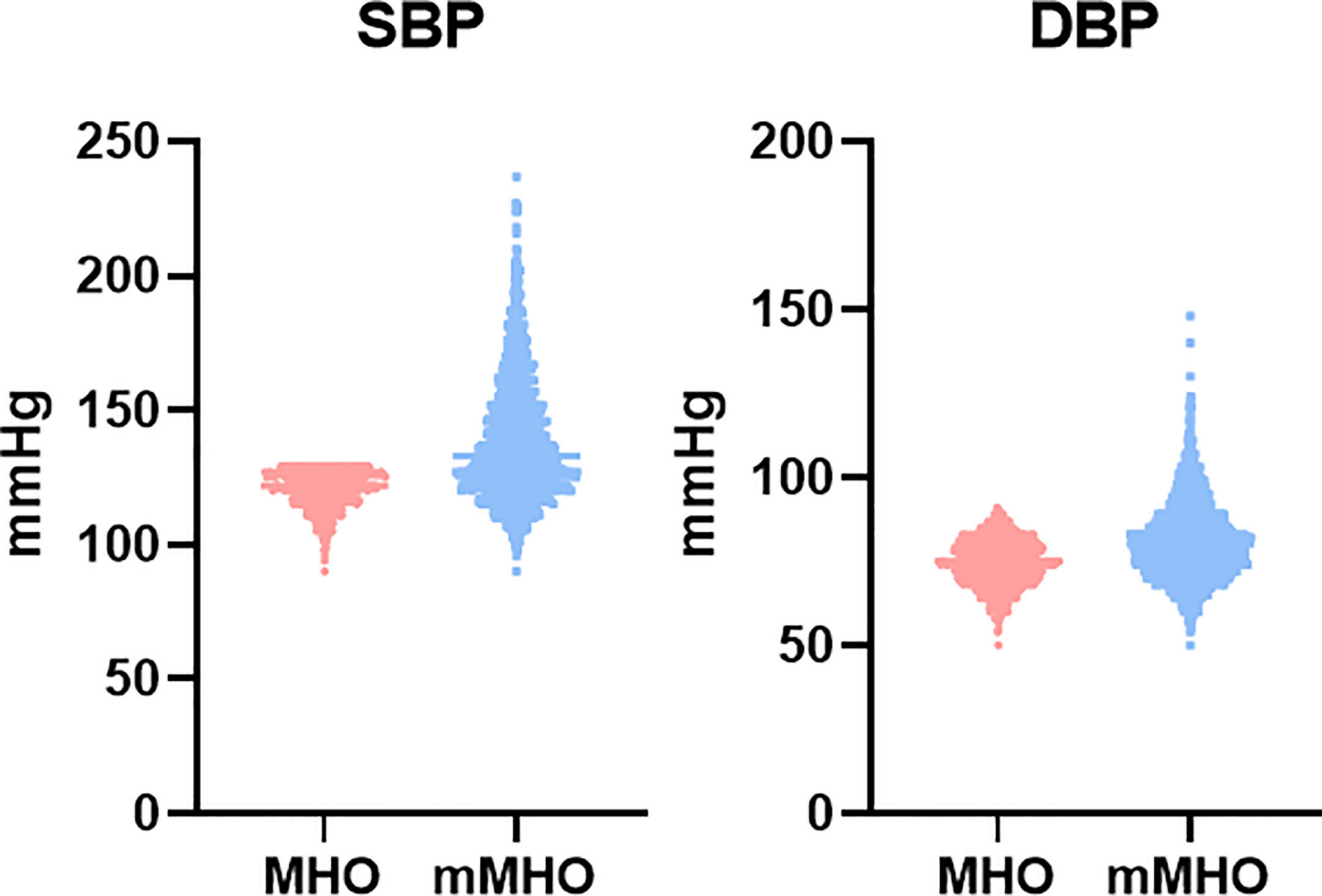
Comparison of BP distribution between MHO groups defined by the two criteria.

By comparing these six metabolic indicators, the results showed that the mMHO group had higher levels of SBP, DBP, and HDL-C, but lower levels of WC, WHR, TG, and FPG (**Table 3**). Among them, except for higher blood pressure, higher HDL-C and lower WC, WHR, TG, FPG are considered to be protective factors for CVD, but the risk of mMHO group was increased instead. We therefore hypothesize that inconsistent blood pressure levels between the two MHO groups may contribute to the different cardiovascular risks, and show this difference by plotting scatter plots, as shown in **Figure 6**. The data showed that the mMHO group had higher overall blood pressure than the MHO group, and included individuals with extremely high blood pressure values.

**Table 3:** Comparison of differences in metabolic indices between MHO and mMHO groups.

	MHO’ (n=249)	mMHO’ (n=893)	MHO&mMHO (n=637)	p-value*
WC (cm)	89.2 ± 6.0	85.8 ± 8.3	85.5 ± 7.4	<0.001
WHR	0.89 ± 0.05	0.87 ± 0.07	0.86 ± 0.06	<0.001
SBP (mmHg)	121 ± 6	151 ± 20	120 ± 7	<0.001
DBP (mmHg)	77 ± 7	86 ± 11	73 ± 6.5	<0.001
TG (mmol/L)	1.3 ± 0.9	1.1 ± 0.5	1.3 ± 0.9	<0.001
HDL-C (mmol/L)	1.1 ± 0.2	1.6 ± 0.3	1.4 ± 0.3	<0.001
FPG (mmol/L)	5.7 ± 0.5	5.4 ± 1.2	5.3 ± 0.5	<0.001

MHO ‘: subjects diagnosed with MHO only according to the new criterion; MHO ‘: subjects diagnosed with MHO only according to traditional criterion; MHO&mMHO: subjects diagnosed with MHO according to both new and traditional criteria.

*p-Value represents the significance of the difference among the three groups in the above table in the Variance analysis and between MHO and mMHO in the post-hoc analysis, and the results are consistent, both < 0.001

## Discussion

This study used a new definition of metabolically healthy obesity to determine the risk of incident cardiovascular disease and mortality among MHO subjects in a cohort study of 9,345 subjects in rural northeastern China during a 4.66-year follow-up period. It was concluded that MHO subjects defined using the new criterion did not experience an increased risk of combined CVD and stroke, whereas metabolically unhealthy subjects, whether with obesity or not, had varying levels of increased risk. At the same time, the present study unexpectedly found an increased risk of CHD in subjects with MHO, which may indicate that obesity plays a more important role than metabolic factors in the development of CHD (31).

“Metabolically healthy obesity” is a very interesting concept that has been widely discussed by researchers. To date, however, the question of increased cardiovascular risk in the MHO population remains controversial. An increasing number of researchers have questioned the concept of MHO after fully assessing the cardiovascular risk of MHO subjects, arguing that the MHO statement is misleading (32, 33). For example, a meta-analysis of eight cohort studies concluded that all people with obesity, even in the absence of metabolic abnormalities, have an increased risk of cardiovascular disease and death compared with MHN individuals, suggesting that there is no healthy pattern of weight gain (13). Another meta-analysis by Fan et al., which defined MHO as absence of metabolic syndrome or insulin resistance, showed an increased risk of both combined CVD and all-cause mortality in healthy overweight and healthy subjects with obesity, also refuting the notion that MHO is truly healthy (15). In addition, there is other evidence that individuals with MHO may be at higher risk in other ways (12, 14, 34, 35). However, several studies have come to different conclusions. For example, the study by Hamer M et al. found no increased risk of cardiovascular disease in MHO participants over a 7-year period after a long-term follow-up study of more than 20,000 people (36).

Due to the lack of consistent diagnostic criteria for metabolic health, various studies have used different criteria to define MHO, resulting in large differences in MHO population characteristics and inconsistent relationship between MHO and cardiovascular risk (17–19). Most studies define metabolic health as having 0-2 metabolic syndrome components, which may arrive at biased results. Among the several factors included in metabolic syndrome, the attributable risks for the occurrence of CVD are different, so there are large characteristics differences among metabolically healthy individuals with 0, 1, or 2 components (34). In addition, several studies have attempted to find new criteria to define MHO in order to identify truly cardiovascular low-risk individuals from populations with obesity. For example, some studies have defined MHO based on homeostasis model assessment of insulin resistance (HOMA-IR) (37–40), exercise testing (41, 42), or a combination of multiple metabolic health criteria (43, 44). However, the results of most studies have been negative.

​Based on the above discussion in this paper, we began to wonder if metabolically healthy obesity is a paradox, and if people with obesity are all at higher cardiovascular risk. We believe the answer is no. In our view, truly healthy individuals with obesity exist, and the key is how to accurately identify them. MHO is not a simple concept that combines metabolic health and obesity, and researchers need more scientific criteria to effectively distinguish the truly at-risk individuals in the populations with obesity. This study is the first to apply the novel metabolic health criterion to an Asian population and demonstrate its predictive value for CVD incidence. More importantly, unlike most previous studies, this study demonstrated that individuals with MHO identified in this way did not have an increased risk of CVD and stroke combined.

At the same time, this study performed a further analysis in this study using two different criteria to define metabolic health in the same cohort and found that MHO was not associated with an increased risk of CVD when using the new criteria. However, mMHO individuals showed a higher risk when the traditional definition criterion was used. A further analysis of MHO subgroups defined according to these two criteria is performed in this study. Unexpectedly, mMHO individuals defined by conventional criteria had better metabolic status (higher HDL-C and lower FPG, TG, WC and WHR) except for blood pressure, but was associated with worse cardiovascular outcomes. By comparing the differences in metabolic indicators between the two groups, it suggests that blood pressure may be a major factor in the relationship between MHO and CVD events. Both include blood pressure as one of the criteria for diagnosis, but the new one includes blood pressure as one of the necessary conditions for diagnosis and imposes stricter requirements. It is possible that the MHO population was erroneously expanded to include subjects with extremely high blood pressure when using traditional criteria to define MHO. In addition, the diagnostic criteria for MHO proposed by other studies, such as the insulin resistance index and the exercise test, also struggle to play a larger role in practical applications due to their measurement procedures and difficulty in obtaining data.​

​ Finally, there are some limitations and shortcomings in the present study. First, the study did not obtain results for fatal CVD and all-cause mortality, which may be explained by the following: 1, the 4.66-year follow-up period was not long enough, and the mortality risk of the subjects in the four groups was not sufficiently exposed. 2, the Univariate Cox Regression results showed that the risk of cardiovascular death and all-cause death was lower in the MHO group and higher in the MUN and MUO groups, with significant differences between the groups. However, after adjusting for age, sex and ethnicity in Cox regression model 1, the difference disappears. By comparing the differences in baseline characteristics between the four groups, the study found that the age and proportion of males in the MUN and MUO groups were significantly higher than those in the MHN and MHO groups. We analyzed that age and gender may have a decisive effect on mortality outcomes, so the effect of metabolic health and obesity status on mortality outcomes becomes insignificant after adjusting for age and gender. Second, neither metabolic health nor obesity status is a steady state and may change over time, and the effect of transitions in metabolic health and obesity status on the occurrence of outcome events was not considered. These deficiencies need to be remedied by longer follow-up and further study.

## Conclusions

Existence of a population of metabolically healthy individuals with obesity with no increased cardiovascular risk. In contrast to traditional criterion, the novel criterion can effectively identify truly low CVD risk individuals among individuals with obesity, which is important for obesity and cardiovascular prevention. The MHO subjects defined by the novel criterion have lower blood pressure compared to the traditional criterion, which may be a key factor in whether individuals with obesity develop CVD. We also call on people with obesity to pay more attention to their metabolic status, rather than obesity itself, and to actively manage on blood pressure.

## Data availability statement

The raw data supporting the conclusions of this article will be made available by the authors, without undue reservation.

## Ethics statement

The Ethics Committee of China Medical University has approved our project (Shenyang, China, ethical approved project identification code: AF-SOP-07-1, 0-01). Every participant received and signed a paper vision informed consent after clarifying relevant information writing with the study objectives, benefits, medical procedures, confidentiality, agreement on personal information, and agreement on publication of related data research.

## Author contributions

XZ and YS directed the design of study. XG were responsible for the study conduct. QL and RM analyzed the data. QL and PW wrote the manuscript. All authors contributed to the article and approved the submitted version.

## References

[1] World Health Organization. Obesity and overweight. Available at: https://www.who.int/news-room/fact-sheets/detail/obesity-and-overweight (Accessed March 20, 2022).

[2] Roth GA Mensah GA Johnson CO Addolorato G Ammirati E Baddour LM. Global burden of cardiovascular diseases and risk factors, 1990-2019: update from the GBD 2019 study. J Am Coll Cardiol (2020) 76(25):2982–3021. doi: 10.1016/j.jacc.2020.11.010 33309175 PMC7755038

[3] Sharma AM. M, m, m & m: a mnemonic for assessing obesity. Obes Rev (2010) 11(11):808–9. doi: 10.1111/j.1467-789X.2010.00766.x 21182728

[4] Berrington de Gonzalez A Hartge P Cerhan JR Hannan L MacInnis RJ Moore SC. Body-mass index and mortality among 1.46 million white adults. N Engl J Med (2010) 363(23):2211–9. doi: 10.1056/NEJMoa1000367 PMC3066051 21121834

[5] Arnlöv J Ingelsson E Sundström J Lind L. Impact of body mass index and the metabolic syndrome on the risk of cardiovascular disease and death in middle-aged men. Circulation (2010) 121(2):230–6. doi: 10.1161/CIRCULATIONAHA.109.887521 20038741

[6] Meigs JB Wilson PW Fox CS Vasan RS Nathan DM Sullivan LM. Body mass index, metabolic syndrome, and risk of type 2 diabetes or cardiovascular disease. J Clin Endocrinol Metab (2006) 91(8):2906–12. doi: 10.1210/jc.2006-0594 16735483

[7] Kissebah AH Vydelingum N Murray R Evans DJ Hartz AJ Kalkhoff RK. Relation of body fat distribution to metabolic complications of obesity. J Clin Endocrinol Metab (1982) 54(2):254–60. doi: 10.1210/jcem-54-2-254 7033275

[8] Sims EAH. Characterization of the syndromes of obesity. In: Brodoff BN Bleicher SJ, editors. Diabetes mellitus and obesity. Baltimore, MD: Williams & Wilkins (1982). p. 219–26.

[9] Mirzaei B Abdi H Serahati S Barzin M Niroomand M Azizi F. Cardiovascular risk in different obesity phenotypes over a decade follow-up: Tehran lipid and glucose study. Atherosclerosis (2017) 258:65–71. doi: 10.1016/j.atherosclerosis.2017.02.002 28213199

[10] Liu JT Yao HY Yu SC Liu JJ Zhu GJ Han SM. Joint association of metabolic health and obesity with ten-year risk of cardiovascular disease among Chinese adults. BioMed Environ Sci (2022) 35(1):13–21. doi: 10.3967/bes2022.003 35078558

[11] Opio J Croker E Odongo GS Attia J Wynne K McEvoy M. Metabolically healthy overweight/obesity are associated with increased risk of cardiovascular disease in adults, even in the absence of metabolic risk factors: a systematic review and meta-analysis of prospective cohort studies. Obes Rev (2020) 21(12):e13127. doi: 10.1111/obr.13127 32869512

[12] Stefan N Häring HU Schulze MB. Metabolically healthy obesity: the low-hanging fruit in obesity treatment? Lancet Diabetes Endocrinol (2018) 6(3):249–58. doi: 10.1016/S2213-8587(17)30292-9 28919065

[13] Kramer CK Zinman B Retnakaran R. Are metabolically healthy overweight and obesity benign conditions?: a systematic review and meta-analysis. Ann Intern Med (2013) 159(11):758–69. doi: 10.7326/0003-4819-159-11-201312030-00008 24297192

[14] Eckel N Meidtner K Kalle-Uhlmann T Stefan N Schulze MB. Metabolically healthy obesity and cardiovascular events: a systematic review and meta-analysis. Eur J Prev Cardiol (2016) 23(9):956–66. doi: 10.1177/2047487315623884 26701871

[15] Fan J Song Y Chen Y Hui R Zhang W. Combined effect of obesity and cardio-metabolic abnormality on the risk of cardiovascular disease: a meta-analysis of prospective cohort studies. Int J Cardiol (2013) 168(5):4761–8. doi: 10.1016/j.ijcard.2013.07.230 23972953

[16] Schulze MB. Metabolic health in normal-weight and obese individuals. Diabetologia (2019) 62(4):558–66. doi: 10.1007/s00125-018-4787-8 30569272

[17] Rey-López JP de Rezende LF Pastor-Valero M Tess BH. The prevalence of metabolically healthy obesity: a systematic review and critical evaluation of the definitions used. Obes Rev (2014) 15(10):781–90. doi: 10.1111/obr.12198 25040597

[18] Hinnouho GM Czernichow S Dugravot A Batty GD Kivimaki M Singh-Manoux A. Metabolically healthy obesity and risk of mortality: does the definition of metabolic health matter? Diabetes Care (2013) 36(8):2294–300. doi: 10.2337/dc12-1654 PMC3714476 23637352

[19] Liu C Wang C Guan S Liu H Wu X Zhang Z. The prevalence of metabolically healthy and unhealthy obesity according to different criteria. Obes Facts (2019) 12(1):78–90. doi: 10.1159/000495852 30814477 PMC6465689

[20] Zembic A Eckel N Stefan N Baudry J Schulze MB. An empirically derived definition of metabolically healthy obesity based on risk of cardiovascular and total mortality. JAMA Netw Open (2021) 4(5):e218505. doi: 10.1001/jamanetworkopen.2021.8505 33961036 PMC8105750

[21] Li Z Guo X Zheng L Yang H Sun Y. Grim status of hypertension in rural China: results from northeast China rural cardiovascular health study 2013. J Am Soc Hypertens (2015) 9(5):358–64. doi: 10.1016/j.jash.2015.02.014 25863573

[22] Li Z Guo X Zheng L Sun Z Yang H Sun G. Prehypertension in rural northeastern China: results from the northeast China rural cardiovascular health study. J Clin Hypertens (Greenwich) (2014) 16(9):664–70. doi: 10.1111/jch.12378 PMC8031851 25131567

[23] Levey AS Stevens LA Schmid CH Zhang YL Castro AF 3rd Feldman HI. A new equation to estimate glomerular filtration rate. Ann Intern Med (2009) 150(9):604–12. doi: 10.7326/0003-4819-150-9-200905050-00006 PMC2763564 19414839

[24] World Health Organization Regional Office for the Western Pacific. The Asia-pacific perspective:Redefining obesity and its treatment (2000). Available at: https://apps.who.int/iris/handle/10665/206936 (Accessed January 5, 2022).

[25] Association. AD. 2. classification and diagnosis of diabetes*: standards of medical care in diabetes-2019* . Diabetes Care (2019) 42(Suppl 1):S13–s28. doi: 10.2337/dc19-S002 30559228

[26] Alberti KG Eckel RH Grundy SM Zimmet PZ Cleeman JI Donato KA. Harmonizing the metabolic syndrome: a joint interim statement of the international diabetes federation task force on epidemiology and prevention; national heart, lung, and blood institute; American heart association; world heart federation; international atherosclerosis society; and international association for the study of obesity. Circulation (2009) 120(16):1640–5. doi: 10.1161/CIRCULATIONAHA.109.192644 19805654

[27] Guo X Li Z Zhou Y Yu S Yang H Sun G. The effects of transitions in metabolic health and obesity status on incident cardiovascular disease: insights from a general Chinese population. Eur J Prev Cardiol (2021) 28(11):1250–8. doi: 10.1177/2047487320935550 34551085

[28] Gaye B Canonico M Perier MC Samieri C Berr C Dartigues JF. Ideal cardiovascular health, mortality, and vascular events in elderly subjects: the three-city study. J Am Coll Cardiol (2017) 69(25):3015–26. doi: 10.1016/j.jacc.2017.05.011 28641790

[29] WHO MONICA project. MONICA manual (1990). Available at: https://www.thl.fi/publications/monica/index.html (Accessed 5 January 2020).

[30] Zhao D Liu J Wang W Zeng Z Cheng J Liu J. Epidemiological transition of stroke in China: twenty-one-year observational study from the sino-MONICA-Beijing project. Stroke (2008) 39(6):1668–74. doi: 10.1161/STROKEAHA.107.502807 18309149

[31] Mann DM Lee J Liao Y Natarajan S. Independent effect and population impact of obesity on fatal coronary heart disease in adults. Prev Med (2006) 42(1):66–72. doi: 10.1016/j.ypmed.2005.09.011 16297443

[32] Zhou Z Macpherson J Gray SR Gill JMR Welsh P Celis-Morales C. Are people with metabolically healthy obesity really healthy? a prospective cohort study of 381,363 UK biobank participants. Diabetologia (2021) 64(9):1963–72. doi: 10.1007/s00125-021-05484-6 PMC8382657 34109441

[33] Lopez-Jimenez F Almahmeed W Bays H Cuevas A Di Angelantonio E le Roux CW. Obesity and cardiovascular disease: mechanistic insights and management strategies. a joint position paper by the world heart federation and world obesity federation. Eur J Prev Cardiol (2022) 29(17):2218–37. doi: 10.1093/eurjpc/zwac187 36007112

[34] Wang W He J Hu Y Song Y Zhang X Guo H. Comparison of the incidence of cardiovascular diseases in weight groups with healthy and unhealthy metabolism. Diabetes Metab Syndr Obes (2021) 14:4155–63. doi: 10.2147/DMSO.S330212 PMC8491784 34621129

[35] Huang MY Wang MY Lin YS Lin CJ Lo K Chang IJ. The association between metabolically healthy obesity, cardiovascular disease, and all-cause mortality risk in Asia: a systematic review and meta-analysis. Int J Environ Res Public Health (2020) 17(4):1320. doi: 10.3390/ijerph17041320 32092849 PMC7068615

[36] Hamer M Stamatakis E. Metabolically healthy obesity and risk of all-cause and cardiovascular disease mortality. J Clin Endocrinol Metab (2012) 97(7):2482–8. doi: 10.1210/jc.2011-3475 PMC3387408 22508708

[37] Smith GI Mittendorfer B Klein S. Metabolically healthy obesity: facts and fantasies. J Clin Invest (2019) 129(10):3978–89. doi: 10.1172/JCI129186 PMC6763224 31524630

[38] Bo S Musso G Gambino R Villois P Gentile L Durazzo M. Prognostic implications for insulin-sensitive and insulin-resistant normal-weight and obese individuals from a population-based cohort. Am J Clin Nutr (2012) 96(5):962–9. doi: 10.3945/ajcn.112.040006 23034958

[39] Kuk JL Ardern CI. Are metabolically normal but obese individuals at lower risk for all-cause mortality? Diabetes Care (2009) 32(12):2297–9. doi: 10.2337/dc09-0574 PMC2782994 19729521

[40] Durward CM Hartman TJ Nickols-Richardson SM. All-cause mortality risk of metabolically healthy obese individuals in NHANES III. J Obes (2012) 2012:460321. doi: 10.1155/2012/460321 23304462 PMC3523154

[41] Farrell SW Fitzgerald SJ McAuley PA Barlow CE. Cardiorespiratory fitness, adiposity, and all-cause mortality in women. Med Sci Sports Exerc (2010) 42(11):2006–12. doi: 10.1249/MSS.0b013e3181df12bf 20351588

[42] McAuley PA Kokkinos PF Oliveira RB Emerson BT Myers JN. Obesity paradox and cardiorespiratory fitness in 12,417 male veterans aged 40 to 70 years. Mayo Clin Proc (2010) 85(2):115–21. doi: 10.4065/mcp.2009.0562 PMC2813818 20118386

[43] Choi KM Cho HJ Choi HY Yang SJ Yoo HJ Seo JA. Higher mortality in metabolically obese normal-weight people than in metabolically healthy obese subjects in elderly koreans. Clin Endocrinol (Oxf) (2013) 79(3):364–70. doi: 10.1111/cen.12154 23330616

[44] Ortega FB Lee DC Katzmarzyk PT Ruiz JR Sui X Church TS. The intriguing metabolically healthy but obese phenotype: cardiovascular prognosis and role of fitness. Eur Heart J (2013) 34(5):389–97. doi: 10.1093/eurheartj/ehs174 PMC3561613 22947612

