# Supplementary material for: A novel criterion of metabolically healthy obesity could effectively identify individuals with low cardiovascular risk among Chinese cohort
Source: Front Endocrinol (Lausanne). 2023 May 26;14:1140472. doi: 10.3389/fendo.2023.1140472 (PMC10273263; doi:10.3389/fendo.2023.1140472)
Supplement: Supplementary file 1 [file Table_1.docx]

**Supplementary Table 1** Baseline characteristics of the four groups of subjects grouped according to traditional criterion for diagnosis of metabolic health

|  | mMHN  (n=4119) | mMUN  (n=1082) | mMHO  (n=1530) | mMUO  (n=2614) | p-Value |
| --- | --- | --- | --- | --- | --- |
| age(year) | 52.7±10.6 | 56.8±10.3 | 50.6±9.6 | 53.9±10.0 | ＜0.001 |
| male(%) | 2081(50.5) | 389(36.0) | 730(47.7) | 1184(45.3) | ＜0.001 |
| ethnicity of Han(%) | 3902(94.7) | 1028(95.0) | 1410(92.2) | 2466(94.3) | 0.034 |
| family income＞5000CNY(%) | 3639(88.3) | 932(86.1) | 1395(91.2) | 2349(89.9) | ＜0.001 |
| family history of CHD(%) | 536(13.0) | 141(13.0) | 216(14.1) | 387(14.8) | 0.17 |
| family history of Stroke(%) | 606(14.7) | 187(17.3) | 249(16.3) | 473(18.1) | 0.002 |
| current smoking(%) | 1696(41.2) | 380(35.1) | 486(31.7) | 795(30.4) | ＜0.001 |
| current drinking(%) | 1049(25.5) | 218(20.1) | 354(23.1) | 589(22.5) | 0.001 |
| regular exercise(%) | 730(17.7) | 263(24.3) | 260(17.0) | 638(32.3) | ＜0.001 |
| BMI(kg/㎡) | 22.0±1.9 | 23.1±1.6 | 27.3±2.8 | 28.3±2.6 | ＜0.001 |
| WC(cm) | 75.1±6.4 | 81.7±6.8 | 85.7±7.9 | 91.0±7.3 | ＜0.001 |
| WHR | 0.82±0.07 | 0.87±0.07 | 0.86±0.07 | 0.90±0.06 | ＜0.001 |
| SBP(mm Hg) | 134±21 | 149±22 | 138±22 | 151±22 | ＜0.001 |
| DBP(mm Hg) | 79±11 | 85±12 | 81±11 | 87±11 | ＜0.001 |
| TC(mmol/L) | 5.0±1.0 | 5.4±1.2 | 5.1±1.0 | 5.5±1.2 | ＜0.001 |
| TG(mmol/L) | 1.1±0.7 | 2.2±2.0 | 1.2±0.7 | 2.3±1.9 | ＜0.001 |
| LDL-C(mmol/L) | 2.7±0.7 | 3.0±0.9 | 3.0±0.8 | 3.2±0.9 | ＜0.001 |
| HDL-C(mmol/L) | 1.6±0.4 | 1.3±0.4 | 1.5±0.3 | 1.2±0.3 | ＜0.001 |
| UA(μmmol/L) | 271±75 | 291±84 | 282±80 | 314±88 | ＜0.001 |
| eGFR(ml/min/1.73㎡) | 95.6±14.7 | 89.7±16.0 | 97.5±13.4 | 91.7±15.5 | ＜0.001 |
| FPG(mmol/L) | 5.5±1.0 | 6.5±2.0 | 5.4±0.9 | 6.4±2.0 | ＜0.001 |
